# Supplementary material for: Feeding practices, dietary adequacy, and dietary diversities among caregivers with under-five children: A descriptive cross-section study in Dodoma region, Tanzania
Source: PLoS One. 2023 Mar 22;18(3):e0283036. doi: 10.1371/journal.pone.0283036 (PMC10032515; doi:10.1371/journal.pone.0283036)
Supplement: Attachment — Submitted filename: Point-by-point response to reviewers.docx [file pone.0283036.s002.docx]

**Feeding practices, dietary adequacy and dietary diversities among caregivers with under-five children: A descriptive cross-section study in Dodoma region, Tanzania**

**Ref: Submission ID:** **PONE-D-22-28886**

**General:** Thank you for submitting your manuscript to PLOS ONE. After careful consideration, we feel that it has merit but does not fully meet PLOS ONE’s publication criteria as it currently stands. Therefore, we invite you to submit a revised version of the manuscript that addresses the points raised during the review process

**JOURNAL REQUIREMENTS**

| **S/N** | **REVIEWER’S COMMENT** | AUTHOR’S RESPONSE | **PG. NUMBER** |
| --- | --- | --- | --- |
| 1 | 1. Please ensure that your manuscript meets PLOS ONE's style requirements, including those for file naming. The PLOS ONE style templates can be found at  <https://journals.plos.org/plosone/s/file?id=wjVg/PLOSOne_formatting_sample_main_body.pdf> and  <https://journals.plos.org/plosone/s/file?id=ba62/PLOSOne_formatting_sample_title_authors_affiliations.pdf> | The manuscript has been revised to meet the PLO ONE’s style requirements | Pg. 1-18 of the revised Manuscript |
|  | Thank you for stating the following financial disclosure:  “NO - This work did not receive any specific grant from funding agencies in the public, commercial, and or non-profit sectors. It was privately sponsored”  At this time, please address the following queries:  **a)** Please clarify the sources of funding (financial or material support) for your study. List the grants or organizations that supported your study, including funding received from your institution. | The funding section has been revised and the financial support information has be made more clearer that the work did not receive any financial or materials support from any financial agencies/organization | Pg. 14 of the revised Manuscript  &  Journal/author’s manuscript submission system |
|  | **b)** State what role the funders took in the study. If the funders had no role in your study, please state: “The funders had no role in study design, data collection and analysis, decision to publish, or preparation of the manuscript.” | The funding section has been revised and made more clearer that “Since no funder was involved in this study, no role concerning them has been provided” | Pg. 14 of the revised Manuscript  &  Journal/author’s manuscript submission system |
|  | **c)** If any authors received a salary from any of your funders, please state which authors and which funders. | The funding section has been revised and made more clearer that “No any authors received a salary from any of your funders, please state which authors and which funders” | Pg. 14 of the revised Manuscript  &  Journal/author’s manuscript submission system |
|  | **d)** If you did not receive any funding for this study, please state: “The authors received no specific funding for this work.”  Please include your amended statements within your cover letter; we will change the online submission form on your behalf | The funding section has been revised and made clearer that ““The authors received no specific funding for this work.” | Pg. 14 of the revised Manuscript  &  Journal/author’s manuscript submission system |
| 2 | Please amend the manuscript submission data (via Edit Submission) to include author “Rabia K. ABDALLAH1” | The name “Rabia K. Abdallah” has been revised and removed as she’s not part of this work and thus, authors declare that it was a typing error. | Pg. 1 (Title page) of the revised Manuscript  &  Journal/author’s manuscript submission system |
|  | Please amend your authorship list in your manuscript file to include author Bariki A. Ambrose | The name “Rabia K. Abdallah” has been revised and removed as she’s not part of this work and thus, authors declare that it was a typing error. | Pg. 1 (Title page) of the revised Manuscript  &  Journal/author’s manuscript submission system |
| 3 | Please include your full ethics statement in the ‘Methods’ section of your manuscript file. In your statement, please include the full name of the IRB or ethics committee who approved or waived your study, as well as whether or not you obtained informed written or verbal consent. If consent was waived for your study, please include this information in your statement as well. | The research ethics part in the methods section has been revised and the name of the IRRC mentioned, informed consent statement added accordingly | Pg. 14 of the revised Manuscript |

**REVIEWER #1: COMMENTS TO THE AUTHOR**

| **S/N** | **REVIEWER’S COMMENT** | AUTHOR’S RESPONSE | **PG. NUMBER** |
| --- | --- | --- | --- |
| 1 | Perception cannot be quantified, Qualitative data collection is best to study perception | The word “perceived” has been revised and removed because the study did not assess participants’ perception of feeding but, their practices on it. Authors’ declare that the word was misused | Pg. 1 – 14 of the revised Manuscript |
| 2 | Wrong order of authors at line 4 | The order of authors has been revised and re-ordered accordingly | Pg. 14 of the revised Manuscript  &  Journal/author’s manuscript submission system |
| 3 | No sampling procedure and sample size determination formula indicated | Sample size and sampling procedures have been revised and added/indicated accordingly | Pg. 7 & 8 of the revised Manuscript |
| 4 | Finding at abstract line 42, 43 contradict that at result in line 229 | Findings in the abstract have been revised so that they tally with those in the result section | Pg. 2 of the revised Manuscript |
| 5 | Result is inadequate due to inefficiency of data collection tools. No evidence for line 320, 321 findings | Authors acknowledge the comment. The methods and results section has been revised and improved accordingly. However, findings presented in this work addressed the variables under study only because findings of this study serve as descriptive baseline observations to the planned intervention and thus, they may be seen inadequate | Pg. 7-22 of the revised Manuscript |
| 6 | Recommendation- No new innovative ideas generated by the research | The recommendation section has been revised and re-written to reflect and demonstrate what has been found in the current study based on the variables under study | Pg. 23 of the revised Manuscript |
| 7 | Some findings are misplaced after reference | The findings and the reference sections have been revised and improved accordingly so that no findings are misplaced after reference | Pg. 12-32 of the revised Manuscript |

**REVIEWER #2: COMMENTS TO THE AUTHOR**

**General comments**

This is an interesting study that provides useful insights into perceived feeding practices, dietary adequacy and dietary diversities among caregivers with under-five children. The findings of the research are clearly presented and well-discussed. Comments have been made to improve the final version.

| **S/N** | **REVIEWER’S COMMENT** | AUTHOR’S RESPONSE | **PG. NUMBER** |
| --- | --- | --- | --- |
| **Abstract** | Line 43: “A 90.7% of children …” is ambiguous. Sentence should be rephrased | Line 43 has been revised and improved accordingly | Pg. 2 of the revised Manuscript |
|  | Line 47 – 50: Rephrase the first sentence to ensure clarity of message. It is lengthy | Line 47-50 have been revised and improved accordingly to ensure clarity as suggested | Pg. 3 of the revised Manuscript |
|  | Line 52/53: Arrange keywords in alphabetical order | Line 52/53 have been revised and the keywords arranged alphabetically | Pg. 3 of the revised Manuscript |
| **Introduction** | Line 79: and/or | Line 79 has been revised and improved accordingly | Pg. 4 of the revised Manuscript |
| **Methods** | Well-written | Authors acknowledges the reviewer’s comment about the methods and materials section | Pg. 6-12 of the revised Manuscript |
| **Results** | Line 207, 217: There should be unit for the (mean) age i.e., years | Line 207, 2017 have been revised and the unit for age written accordingly | Pg. 12 of the revised Manuscript |
|  | Line 209/210, 219: “A 68.2% …” is ambiguous. Sentence is confusing and should be rephrased | Line 209/210, 2019 have been revised and the sentence re-written accordingly | Pg. 12 of the revised Manuscript |
|  | Table 1 (2nd Row), Table 2 (5th Row), Table 4 (5th Row): Is that the mean age? If it is, it should be specified | Table 1 (2nd Row), Table 2 (5th Row), Table 4 (5th Row) have been revised and specified that in they are mean ages respectively | Pg. 13, 14, & 19 of the revised Manuscript |
|  | Line 252: Preferably, do not commence sentences with figures written as numbers | Line 252 has been revised and re-written accordinlgy | Pg. 12 - 19 of the revised Manuscript |
|  | Table 3, Table 4: P-values which are statistically significant should be signified | p-values in Table 3 and 4 have been revised and signified | Pg. 17-19 of the revised Manuscript |
| **Discussion** | Well-written | Authors acknowledges the reviewer’s comment about the methods and materials section | Pg. 19-22 of the revised Manuscript |
| **Conclusion** | The Conclusion should be specific and straightforward. It should provide answers to the main objective of the study. It should be rewritten | The conclusion section has been revised and re-written accordingly | Pg.22 of the revised Manuscript |
|  | Line 334: Recommendations | The recommendation section has been revised and re-written accordingly | Pg.23 of the revised Manuscript |
|  | Line 345 – 349: Rephrase. Sentence is too long | Line 345-349 have been revised and rephrased accordingly | Pg. 23 of the revised Manuscript |

**REVIEWER #3: COMMENTS TO THE AUTHOR**

**General Comments:** The background created a gap, but the study is a bit complex. First, perception in this study is not well understood. How was perception measured? All I can deduce is feeding practices of caregivers. So, the study should be on “Feeding practices, dietary adequacy and dietary diversities among caregivers with under-five children: A descriptive cross-sectional study in Dodoma region, Tanzania” if the present study would remain as it is. Second, two manuscripts could emerge from this study to make each sharper and simple. For example, one could be on “Feeding practices and dietary diversities among caregivers with under-five children” and the second could be on “dietary adequacy”. I strongly recommend this split.

| **S/N** | **REVIEWER’S COMMENT** | AUTHOR’S RESPONSE | **PG. NUMBER** |
| --- | --- | --- | --- |
| **General comments** | First, perception in this study is not well understood. How was perception measured? | The word “perceived” has been revised and removed because the study did not assess participants’ perception of feeding but, their practices on it. Authors’ declare that the word was misused | Pg.1- 23 of the revised Manuscript |
|  | So, the study should be on “Feeding practices, dietary adequacy and dietary diversities among caregivers with under-five children: A descriptive cross-sectional study in Dodoma region, Tanzania” if the present study would remain as it is | The study title has been revised and the word “perceived/perception” omitted as suggested | Pg. 1 of the revised Manuscript |
|  | Two manuscripts could emerge from this study to make each sharper and simple. For example, one could be on “Feeding practices and dietary diversities among caregivers with under-five children” and the second could be on “dietary adequacy”. I strongly recommend this split | Authors have acknowledged the reviewers recommendation on the split of two manuscripts from this study. However, based on the ethical clearances of the institution and being the baseline findings for the coming interventional study and avoid the repetition of the concepts/context we would advise it remain in its current state as it is | Pg. 1-32 of the revised Manuscript |
| **Methods** | There should be a total population from where the sample was drawn:   - What is the population of under-five children in the study area? - Then, how was the sample size calculated? - What were the sampling procedure and techniques used? | Sample size and sampling procedures have been revised and the details about them added/indicated accordingly | Pg. 7-8 of the revised Manuscript |
|  | It is not also clear whether the respondents (caregivers) were met in their homes or in common venue. For example, this sentence “Caregivers were reached at their homes and data collection procedures were performed by the principal investigator and trained assistant researchers in an unoccupied venue available at the respective ward to assure privacy” is not clear | The data collection procedures section has been revised and re-written to improve clarity as suggested | Pg. 10 of the revised Manuscript |
| **Results** | The results section is cumbersome (four Tables and five figures) and that’s more reason to split the manuscript into two. This will affect the discussion | The result and discussion sections have been revised so that they tally in a simple and clear way. Dietary adequacy and diversifications have been treated in this study as the patterns/characterization of feeding practices. The discussion has also revised to demonstrate the key findings on feeding practices alongside its characterization and determinants | Pg. 12-22 of the revised Manuscript |

**REVIEWER #4: COMMENTS TO THE AUTHOR**

Thanks for the opportunity to review this paper titled: “Perceived feeding practices, dietary adequacy and dietary diversities among caregivers with under-five children: A descriptive cross-section study in Dodoma region, Tanzania” This addresses an important issue in public health, however, the quality of the paper is marred by so many issues (including grammatical errors) which the authors need to address. For instance, there are so many verb agreement and punctuation mistakes throughout the manuscript, and I have tried to indicate some of them. Also, the paper requires major English editing. Therefore, the authors should subscribe to an editing service for adequate and professional editing of this paper

| **S/N** | **REVIEWER’S COMMENT** | AUTHOR’S RESPONSE | **PG. NUMBER** |
| --- | --- | --- | --- |
| **General comments** | The quality of the paper is marred by so many issues (including grammatical errors) which the authors need to address. For instance, there are so many verb agreement and punctuation mistakes throughout the manuscript, and I have tried to indicate some of them. Also, the paper requires major English editing. Therefore, the authors should subscribe to an editing service for adequate and professional editing of this paper | The manuscript has been revised and improved for the grammatical errors and English editing | Pg. 1-32 of the revised Manuscript |
| **Abstract** | The abstract should be presented in a clearer manner with more findings included in the result subsection | The abstract has been revised and the result section improved accordingly | Pg. 2 of the revised Manuscript |
|  | Line 29: ‘Feeding newborn while at health facilities assure…’(there is a verb agreement mistake in this clause) | Line 29 has been revised and improved accordingly to make it clearer | Pg. 2 of the revised Manuscript |
|  | Line 35: (not clear) | Line 35 has been revised and improved accordingly | Pg. 2 of the revised Manuscript |
|  | Line 44: ‘A 90.7% of children were fed group one 44 foods of which 59.1% (n =152) of them were not fed …’(there is an agreement mistakes in this) | Line 44 has been revised and re-written accordingly to make it clearer | Pg. 2 of the revised Manuscript |
|  | Line 66: ‘Proper adherence to dietary adequacy, improvising dietary diversities and proper feeding practices is be’ (verb agreement mistake) | Line 66 has been revised and re-written to make it clearer | Pg. 4 of the revised Manuscript |
| **Introduction** | This needs better organization for smooth flow. The messages are scattered all over the section. It’s good to start with global situation, then African/LMIC and finally Tanzania. Each paragraph should have just one message | The introduction section has been revised and re-written to establish a clear flow of the concepts for easy understanding | Pg. 4-6 of the revised Manuscript |
|  | Line 68: Existing 68 knowledge uncover that (verb agreement mistake) | Line 68 has been revised and re-written accordingly to make it clearer | Pg. 4 of the revised Manuscript |
|  | The authors should make a stronger justification for the paper and show how the paper contributes to new knowledge | Authors have revised the manuscript and try to demonstrate the contribution of the paper that it tries to reveal the unknown about home feeding practices among caregivers | Pg. 1-24 of the revised Manuscript |
| **Methods** | Relevant characteristics of the study area/site are missing in this manuscript | The methods part in the study setting section has been revised and improved accordingly | Pg. 7 of the revised Manuscript |
|  | Considering the sampling technique, more details, such as the sampling interval and sampling ratio should be provided | Sampling procedures section has been revised and more details added to make it clearer | Pg. 7-8 of the revised Manuscript |
|  | How was the sample size calculated, and which software program was used? | Sample size determination section has been revised and more details added to make it clearer | Pg.7-8 of the revised Manuscript |
|  | Write in details about the study participants, selection criteria (inclusion and exclusion) | Participants’ selection criteria has been revised and the section added accordingly | Pg. 8 - 9 of the revised Manuscript |
|  | Were questionnaire structured? | The data collection tools section has been revised and questionnaires have been specified based on the nature of items and administration | Pg. 9 of the revised Manuscript |
| **Results** | The abstract and the methodology indicate that 298 caregivers were sampled and the response rate was 100%, why did the authors report only 289 in the result section? | Authors acknowledge the discrepancy in the sample size between sections. This was the typing error. Sample size in the result section is correct and thus, changes in the abstract and methods sections made accordingly | Pg. 2, & 8 of the revised Manuscript |
|  | The interpretations of the results should be better stated for clarity | The results section has been revised and improved accordingly as suggested | Pg. 12-20 of the revised Manuscript |
| **Discussion** | The discussion is weakly presented and did not address most of the findings in the result section | The discussion section has been revised and re-written to reflect the study findings as suggested | Pg. 20-22 of the revised Manuscript |
|  | There is a mix up of different referencing styles (lines 301-310) | The citation and references have been revised and improved accordingly | Pg. 20-22 of the revised Manuscript |
|  | The authors may consider starting the discussion with a summary statement that showcases the overall message of the study | The discussion section has been revised and improved accordingly to show the overall message of the study as suggested | Pg. 20-22 of the revised Manuscript |
|  | Line 358: apart from addressing a very important issue in maternal and child health, what are the strengths of this study? | The strengths section of the study on hand have been revised and more details added accordingly as suggested | Pg. 25 of the revised Manuscript |
| **Conclusion** | This should be revised and better presented to capture the key findings of the study | The conclusion section has been revised and improved accordingly to reflect the study findings | Pg. 22-23 of the revised Manuscript |
|  | 320: ‘Even though many caregivers are empowered with nutritional knowledge during their reproductive health clinic (RCH) visits and various scholarly…’ this does not appear to be part of the finding of this study | Line 320 has been revised and modified accordingly to reflect the study findings | Pg.22-23 of the revised Manuscript |
| **Ethical approval** | The number of the ethical approval from the Health Research Ethics Committee of the institution should be indicated | The research ethics section has been revised and improved accordingly by a indicating the number of ethical approval from the IRRC | Pg. 25-26 of the revised Manuscript |
