# Supplementary material for: Feeding practices, dietary adequacy, and dietary diversities among caregivers with under-five children: A descriptive cross-section study in Dodoma region, Tanzania
Source: PLoS One. 2023 Mar 22;18(3):e0283036. doi: 10.1371/journal.pone.0283036 (PMC10032515; doi:10.1371/journal.pone.0283036)
Supplement: Attachment — Submitted filename: Point-by-point response to reviewers.docx [file pone.0283036.s003.docx]

**Feeding practices, dietary adequacy and dietary diversities among caregivers with under-five children: A descriptive cross-section study in Dodoma region, Tanzania**

**Ref: Submission ID:** **PONE-D-22-28886R1**

**REVIEWER #2**

**General:** The manuscript should be accepted for publication after the few corrections, stated below, have been addressed.

| **S/N** | **REVIEWER’S COMMENT** | AUTHOR’S RESPONSE | **PG. NUMBER** |
| --- | --- | --- | --- |
| **Abstract** | **Results:** Preferably, do not start sentences with figures written as numbers. Correct this, wherever applicable, in the manuscript. | The result part of the abstract and the rest of the manuscript has been revised not to start sentences with figures/numbers as suggested | Pg. 2-25 of the revised Manuscript |
| **Introduction** | Lines 80, 83, 408: Include a slash “and/or” | Line 80, 83, 408 and the rest of the work has been revised and a slash “and/or” has been included | Pg. 2-25 of the revised Manuscript |
| **Results** | Line 252: Preferably, do not commence sentences with figures written as numbers. | Line 252 have been revised not to start sentences with figures written as numbers as suggested | Pg. 12 of the revised Manuscript |
|  | Table 3, Table 4: keynotes should include “P < 0.05 is statistically significant” | Table 3 and Table 4 have been revised and the key notes have included “p<0.05 is statistically significant as suggested | Pg. 18-20 of the revised Manuscript |
|  | Line 419: “Recommendations” not Recommendation | Line 419 has been revised and the word “Recommendation” added “s” at the end to be “Recommendations” | Pg. 23 of the revised Manuscript |
|  | Line 488: “Acknowledgements” not “Acknowledgement. | Line 488 has been revised and the word “Acknowledgement” has been added “s” to be “Acknowledgements” | Pg. 27 of the revised Manuscript |

**REVIEWER #4**

The authors have tried to improve the general quality of this manuscript in the revised version, and have attended to most of the issues I raised, following my first review of the paper. I commend them. However, I still have a couple of concerns that I would like addressed

| **S/N** | **REVIEWER’S COMMENT** | AUTHOR’S RESPONSE | **PG. NUMBER** |
| --- | --- | --- | --- |
| **Methods** | **Study setting**  **-**This paper focuses on “dietary practices, adequacy and diversities…” among caregivers.  -The high prevalence of stunting in the area was stated, but the authors failed to include such details as climatic conditions/vegetation in relation to food security and the socio-cultural beliefs related to infant and young child feeding, in the description of study setting | The study setting section has been revised and improved accordingly to include and link the climatic condition to food security and socio-cultural beliefs to feeding practices | Pg. 7 of the revised Manuscript |
|  | “The region was sampled owing to the growing prevalence…” -How was it sampled? (Purposively? randomly? Etc.)…this should be under sampling technique | Sampling procedures section has been revised and more details added to make it clearer on how the study setting was sampled | Pg. 8-9 of the revised Manuscript |
|  | **Study population:**  **Inclusion criteria:** “… a resident of Dodoma region for at least more than six months,” -What is your reason for choosing residency period of at least more than six months? | Inclusion criteria of the study population has been revised and the period limit has been removed as it had no any significant rationale | Pg.7-8 of the revised Manuscript |
|  | **Exclusion criteria:** Deaf, dumb, mentally unsound, reported sicknesses.  -What is the rationale for excluding these groups of people, especially the deaf and dumb? I think excluding the deaf and dumb among the study group raises some ethical questions of fairness.  -Kindly explain in details why deaf and dumb made the exclusion criteria. How will you establish those who are mentally unsound? | Participants’ exclusion criteria has been revised and improved accordingly based on ethical issues | Pg. 9 of the revised Manuscript |
